# Leptomeningeal spread in high-grade gliomas: Is surgery or adjuvant therapy after leptomeningeal spread associated with survival benefit?

**DOI:** 10.1007/s10143-023-02209-8

**Published:** 2023-11-23

**Authors:** Shuai Zhong, Xiaojun Fu, Chenxing Wu, Rui Liu, Shouwei Li

**Affiliations:** Department of Neurosurgery, Sanbo Brain Hospital, Capital Medical University, Beijing, China

**Keywords:** Leptomeningeal spread, High-grade glioma, Glioblastoma, Magnetic resonance imaging, Survival, Treatment

## Abstract

**Purpose:**

This study aimed to identify prognostic factors associated with survival in patients with high-grade glioma (HGG) after leptomeningeal spread (LMS) and to clarify the behavior and treatment response.

**Methods:**

This retrospective study included 114 patients with HGGs diagnosed with LMS from August 1, 2014, to July 30, 2021, at our institution. Clinical, radiological, pathological, and outcome data were collected. Univariable and multivariable Cox regression were used for overall survival (OS) and post-LMS survival (PLS) analysis.

**Results:**

The median OS was 17.0 months and the median PLS was 6.0 months. Gross total resection (GTR) after LMS diagnosis and pathology grade III were statistically significantly associated with longer OS in all patients. GTR after LMS diagnosis and nodular LMS were independent favorable prognostic factors on PLS. Non-adjuvant therapy after LMS diagnosis was associated with shorter OS and PLS. In glioblastoma (GBM) subgroup analysis, GTR after LMS diagnosis and secondary LMS were independent favorable prognostic factors on OS. Karnofsky Performance Status (KPS) of ≥80 at LMS diagnosis, chemotherapy after LMS and intrathecal methotrexate (MTX) treatment were statistically significantly associated with longer PLS. MRI type II was a predictor of shorter PLS.

**Conclusion:**

The treatment of patients with glioma after LMS diagnosis is very challenging and limited. Safe GTR of tumor and subsequent adjuvant therapy after LMS remains a powerful weapon to improve survival for HGG patients with LMS. Chemotherapy and Intrathecal MTX treatment are feasible treatments after LMS. The extent of tumor dissemination may affect the survival after LMS.

## Introduction

High-grade gliomas (HGGs) are the most frequent primary malignant tumors in the central nervous system. Unfortunately, the prognosis remains poor with a dismal median overall survival (OS) of 14.0–21.0 months for grade IV tumors despite the use of surgical resection, radiation, chemotherapy, tumor-treating fields, and other treatments [1, 2]. Tumor recurrence and progression often occur in a short time because HGG grows highly invasively [1, 3]. Different progression patterns, including local, distant, diffuse, multifocal progression, and leptomeningeal spread (LMS), have been well established [4–6]. LMS is often ascribed to a worse prognosis than parenchymal progression [7, 8]. LMS becomes more common in our clinical practice with the continuous advancement of treatment and image techniques, but data on LMS in HGG remains scarce [6–9].

LMS was first described in the spinal cord which metastasize from supratentorial glioblastomas (GBM) in 1931 [9]. Currently, LMS is a tumor cell that flows along with cerebrospinal fluid (CSF) to the subarachnoid spaces or ventricle, resulting in an abnormal linear or nodular enhancement in the subarachnoid spaces or the cerebral subependymal zone on magnetic resonance imaging (MRI) [3, 6, 7, 9, 10]. Previous studies revealed that patients with LMS had a worse median OS of 16.7 months than those without LMS at 32.0 months [11]. LMS is considered one of the rare and serious complications, with a median OS of 2–5 months after LMS diagnosis [9]. Some previous reports have revealed various incidence of LMS from 4.0% to 23.4%, which is increasing annually [7, 10, 12]. More frequent LMS testing, MRI resolution improvement, and OS improvement may contribute to the increased incidence of LMS [7, 10, 13].

Currently, a standardized treatment method or consensus is not available for patients with LMS [13]. The treatment for LMS is numerous, including radiotherapy, ventriculoperitoneal (VP) shunt, intrathecal chemotherapeutics, targeted therapy, and immunotherapy, but the therapeutic efficacy is limited [7, 10, 13]. Surgery is usually considered unsuitable [9]. As far as we know, there is currently no strong evidence to support this viewpoint. The surgical treatment and adjuvant therapy of patients with LMS is controversial.

Therefore, we retrospectively collected data from patients with HGG with LMS at our institution and performed a comprehensive analysis of prognostic factors for patients with HGG after LMS diagnosis. This study aimed to describe the clinicopathological features, imaging features, and treatment and determine prognostic factors to clarify the behavior and response to treatment after LMS.

## Materials and methods

### Patients

**Fig. 1:**
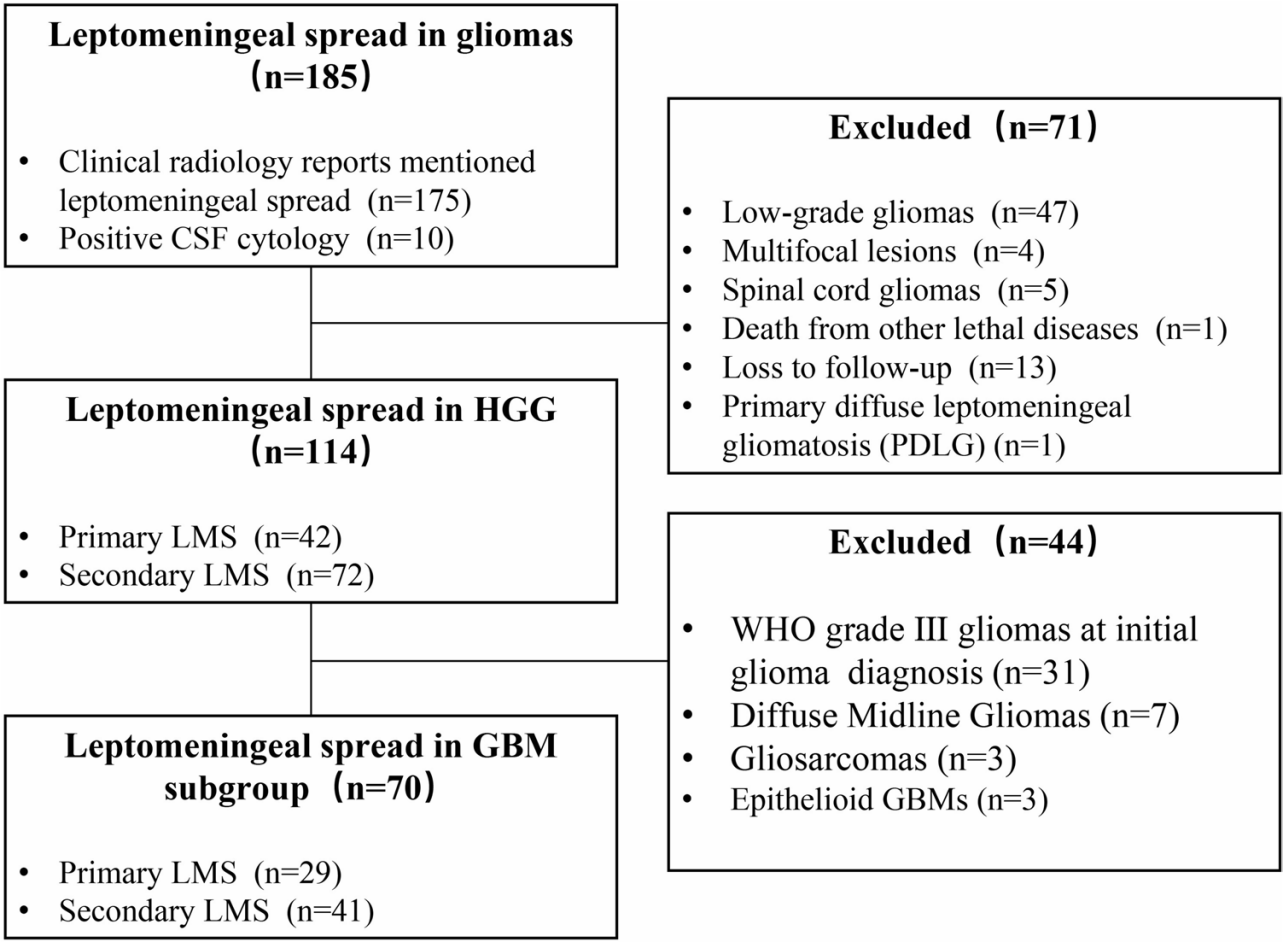
Patient selection flow chart

The Medical Ethics Committee of Capital Medical University approved this study. We retrospectively identified patients with HGG who developed LMS at Sanbo Brain Hospital, Capital Medical University, from August 1, 2014, to July 30, 2021. The inclusion criteria were pathological HGG diagnosis at the initial diagnosis, clinical radiology reports mentioning LMS or subependymal dissemination, or positive CSF cytology in pathology reports. This study excluded patients with low-grade glioma, multifocal lesions, spinal cord glioma, and death from other lethal diseases, as well as patients diagnosed with primary diffuse leptomeningeal gliomatosis. All patients were pathologically confirmed by experienced neuropathologists according to the 2016 World Health Organization classification system when necessary. Finally, 114 patients were included in this study and a GBM subgroup analyses of 70 patients were also performed. An illustration of the workflow with inclusion and exclusion is provided as Fig. 1. Data collected included clinical, radiological, pathological and survival information.

### Radiological evaluation

**Fig. 2:**
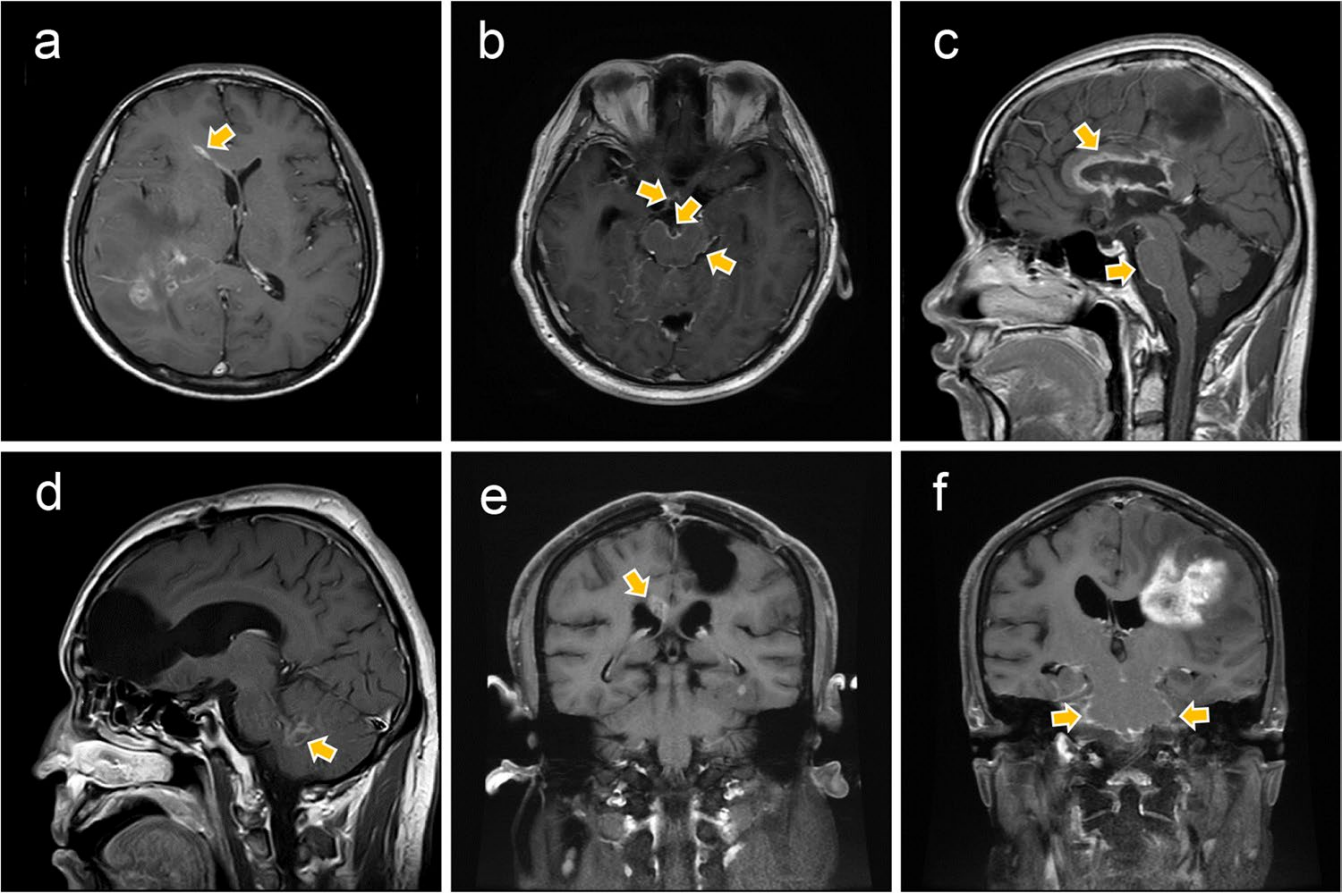
MRIs of HGG patients with leptomeningeal spread. (**a**) MRI type Ia: Axial T1 enhanced-contrast showed line subependymal enhancement of lateral ventricle anterior horn (yellow arrow); (**b**) MRI type Ib: Axial T1 enhanced-contrast showed line leptomeningeal enhancement around midbrain and optic chiasma (yellow arrows); (**c**) MRI type II; Sagittal T1 enhanced-contrast images showed subependymal enhancement along the corpus collosum, line leptomeningeal enhancement in the surface of the brain stem and nodular enhancement in cerebellar tonsil (yellow arrows); (**d**) Nodular LMS: Sagittal T1 enhanced-contrast images showed nodular LMS at the bottom of the fourth ventricle was secondary to frontal lobe glioma (yellow arrow); (**e**) Coronal T1 enhanced-contrast MRI showed stable disease at initial tumor site and contralateral subependymal enhancement (yellow arrow); (**f**) Coronal T1 enhanced-contrast MRI showed local recurrence and leptomeningeal enhancement around brain stem (yellow arrows)

Two well-experienced radiologists independently confirmed LMS on imaging. The LMS was defined as linear or nodular contrast enhancement of the subarachnoid spaces or the cerebral subependymal zone or positive in CSF cytology, which was described in details in a previous study [14]. We defined a new classification pattern according to the location and extent of dissemination. Tumors were classified as type Ia (Fig. 2a) if the contrast-enhancing lesion contacted subependymal zone; Type Ib (Fig. 2b) was classified by enhancement in subarachnoid spaces, including the cerebral gyri and sulci, the cerebellar folia or cortical surface, brainstem or spinal cord surface, and nerve roots or the basal cisterns; Type II (Fig. 2c) was classified by enhancement in both subarachnoid spaces and subependymal zone. The following equation defined the degree of tumor resection: (preoperative tumor volume − postoperative tumor volume)/preoperative tumor volume, as gross total resection (GTR) (>98% resection) and non-GTR (<98%). Nodular LMS was defined as only one nodule enhancement (Fig. 2d). We also considered the presence of a local recurrence from the original tumor burden (Fig. 2e, f) or hydrocephalus, when LMS occurred.

### Statistical analysis

OS was defined as the time from glioma diagnosis to the time of death or last follow-up. Time from LMS diagnosis to death was defined as the post-LMS survival (PLS). Univariable Cox regression analysis was performed for each of the included variables. The multivariate Cox proportional hazards model included variables that reached a significance level of α < 0.1 univariately to identify the factors independently correlated with the survivals. Cox proportional hazard regression model was employed to estimate the hazard ratio (HR) for each potential prognostic factor. Results of interest were graphically presented with Kaplan–Meier curves. Log-rank analysis was used to compare Kaplan–Meier plots. OS data were censored during the last follow-up if the patient was still living. Statistical Package for the Social Sciences version 25.0 (IBM Corporation, Armonk, NY, USA) was used for statistical analysis. Probability values were obtained using two-sided tests with statistical significance defined as P-values of <0.05.

## Results

### Patient characteristics of HGGs with LMS

This study enrolled 114 patients, including 76 (66.7%) males and 38 (33.3%) females with a mean age of 41.5 ± 15.4 years. Patients were followed up for a median time of 16.5 (2.0–117.0) months postoperatively. LMS could occur during glioma diagnosis or recurrence or progression, which was defined as primary LMS and secondary LMS. Primary LMS consisted of 42 (36.8%) patients, including three with anaplastic astrocytomas, two with pleomorphic xanthoastrocytomas, 29 with GBMs, one with epithelioid GBM, five diffuse midline gliomas, and two gliosarcomas. Secondary LMS consisted of 72 (63.2%) patients, including 41 with GBMs, two with diffuse midline gliomas, two with epithelioid GBMs, one with gliosarcoma, five with anaplastic oligodendrogliomas, and 21 with anaplastic astrocytomas at the initial glioma diagnosis. Progression to glioblastoma at LMS diagnosis was confirmed in 14 patients originally diagnosed with anaplastic astrocytoma or oligodendroglioma. Five patients were unable to obtain the latest pathology diagnosis due to lacking surgery or biopsy after LMS diagnosis. Table 1 and 2 respectively shows the clinical, radiological, and pathological characteristics at initial glioma diagnosis and at LMS diagnosis. IDH mutation information was unavailable and pathological tissue was not obtained in six patients because they only received an Ommaya reservoir in our hospital.

**Table 1:** Clinical, radiological and pathological characteristics of patients at initial glioma diagnosis

Variable	At glioma diagnosis(n=114)
Age, Median (Min–Max), y	38.0 (6-80)
Gender	
male	76 (76.7)
female	38 (33.3)
Tumor location	
Supratentorial	101 (88.6)
Infratentorial	13 (11.4)
KPS	83.3±9.2
Extent of resection at glioma diagnosis	
Biopsy	14 (12.3)
Subtotal	48 (42.1)
Gross total resection	52 (45.6)
Pathology	
III	31 (10.1)
IV	83 (89.9)
Adjuvant therapy after gliomas diagnosis	
Radiotherapy	90 (78.9)
Chemotherapy	98 (86.0)
Antiangiogenic therapy	24 (21.1)
Clinical trails	4 (3.5)
Intrathecal MTX	12 (10.5)
Time at LMS diagnosis	
Primary LMS	42 (36.8)
Secondary LMS	72 (63.2)
GTR after LMS diagnosis	27
Non-adjuvant therapy after LMS diagnosis	33 (28.9)
IDH mutation	21/108^a^(19.4)
MGMT methylation	
Yes	6/26^b^(23.1)
No	20/26^b^(76.9)

*IDH* isocitrate dehydrogenase, *MGMT* O6-methylguanine-methyltransferase, *MTX* methotrexate

^a^The IDH status of 6 patients was not available

^b^The MGMT methylation status was available in 26 patients

**Table 2:** Clinical, radiological and pathological characteristics of patients at LMS diagnosis

Variable	At LMS diagnosis(n=114)
Age, Median (Min–Max), y	40.0 (7-80)
Gender	
male	76 (76.7)
female	38 (33.3)
Tumor location	
Supratentorial	97/110^a^(90.4)
Infratentorial	13/110^a^ (9.6)
KPS at LMS diagnosis	79.7±13.5
Extent of resection	
Biopsy	11 (9.6)
Subtotal	43 (37.7)
Gross total resection	27 (23.7)
Ommaya	15 (13.2)
Non operation	21 (18.4)
Pathology	
III	11/109^b^(10.1)
IV	98/109^b^(89.9)
Progression patterns	
Local recurrence +LMS	56/72^c^(77.8)
Simple LMS	16/72^c^(22.2)
MRI characteristics	
Ia	50 (43.9)
Ib	27 (23.7)
II	37 (32.5)
Nodular LMS	18 (15.8)
Adjuvant therapy after LMS diagnosis	
Radiotherapy	27 (23.7)
Chemotherapy	69 (60.5)
Antiangiogenic therapy	23 (20.2)
Intrathecal MTX	12 (10.5)
Non-adjuvant therapy	33 (28.9)
Clinical trails	4 (3.5)
Hydrocephalus	27 (23.7)
IDH mutation	21/108^d^(19.4)
MGMT methylation	
Yes	6/26^e^(23.1)
No	20/26^e^(76.9)

*IDH* isocitrate dehydrogenase, *MGMT* O6-methylguanine-methyltransferase, *MTX* methotrexate

^a^Lesions with a diameter greater than 1cm

^b^The pathological level of 5 patients is unknown

^c^Progression patterns is only assessed in the secondary LMS

^d^The IDH status of 6 patients was not available

^e^The MGMT methylation status was available in 26 patients

### Radiographic characteristics of patients with LMS

According to the radiographic features of dissemination, we categorize it into three types. Brain MRI was obtained in all cases and spinal MRI was obtained in 41 patients. This cohort included 50 (43.9%), 27 (23.7%), and 37 (32.5%) with types Ia, Ib, and II, respectively. Nodular LMS occurred in 18 (15.8%) patients. Secondary LMS was identified in 16 (14.0%) patients with stable disease at the initial tumor site. Hydrocephalus occurred in 27 (23.7%) patients at the time of LMS diagnosis.

### Management of patients after glioma diagnosis and LMS diagnosis

Table 1 outlines the management strategies after glioma diagnosis. All patients underwent surgical intervention at the time of glioma diagnosis, including biopsies in 14 (12.3%), gross total resections in 52 (45.6%), and subtotal resections in 48 (42.1%) patients. Subsequent adjuvant therapy was performed in all patients, included radiotherapy in 90 (78.9%), chemotherapy in 98 (86.0%), intrathecal methotrexate in 12 (10.5%), antiangiogenic therapy in 24 (21.1%) patients, etc. The Stupp protocol was performed on 71 patients. At least two surgical treatments were performed on 73 patients. In the secondary LMS group, 55 (76.4%) cases had ventricular entry during initial resection, 15 (20.8%) had no ventricular entry, and 2 (2.8%) cases had no records.

Table 2 outlines the management strategies after LMS diagnosis. Tumor resection was performed on 70 (61.4%) patients after the LMS diagnosis, among them, 27 (11 with primary lesions, 12 with local recurrent lesions and four with disseminated lesions) underwent GTR. Fifteen (13.2%) patients received the Ommaya reservoir, while only 12 patients underwent subsequent intrathecal methotrexate (MTX) treatment. Operations were not performed on 21 (17.4%) patients who directly underwent subsequent adjuvant treatment. Subsequent adjuvant treatment after LMS diagnosis was administered in 81 (71.1%) patients, including radiotherapy (27, 23.7%), chemotherapy (69, 60.5%), intrathecal MTX (12, 10.5%), antiangiogenic therapy (23, 20.2%), and clinical trials (4, 3.5%). All 27 cases with hydrocephalus underwent VP shunt. Among the patients with secondary LMS, there were 56 patients with local recurrence and LMS.

### Prognostic factors of OS in all patients

Death was recorded in 106 patients upon study completion. The median OS was 17.0 months. Univariate analysis demonstrated statistically significant associations between OS and pathology grade

**Fig. 3:**
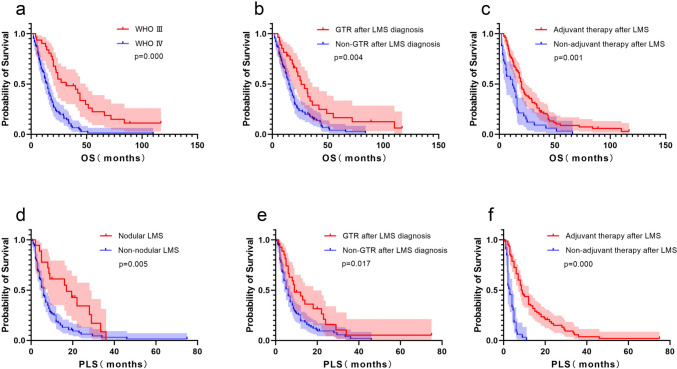
Comparison of OS and PLS by Kaplan–Meier curves in all patients. OS of (**a**) pathology grade, (**b**) GTR after LMS diagnosis and (**c**) non-adjuvant therapy after LMS diagnosis; PLS of (**d**) nodular LMS, (**e**) GTR after LMS diagnosis and (**f**) non-adjuvant therapy after LMS diagnosis

III (HR: 0.343, 95% CI: 0.214-0.548, p = 0.000), KPS of ≥80 (HR: 0.480, 95% CI: 0.287-0.804, p = 0.005), GTR at glioma diagnosis (HR: 0.313, 95% CI: 0.204-0.480, p = 0.000), radiotherapy (HR: 0.354, 95% CI: 0.217-0.577, p = 0.000), chemotherapy (HR: 0.373, 95% CI: 0.214-0.651, p = 0.001), primary LMS (HR: 3.147, 95% CI: 2.046-4.841, p = 0.000), GTR after LMS diagnosis (HR: 0.511, 95% CI: 0.317-0.823, p = 0.006), non-adjuvant therapy after LMS diagnosis (HR: 2.044, 95% CI: 1.346-3.105, p = 0.001), IDH mutation (HR: 0.285, 95% CI: 0.162-0.501, p = 0.000), MGMT methylation (HR: 0.220, 95% CI: 0.062-0.788, p = 0.020) (Table 3). Multivariable analysis revealed that pathology grade III (HR: 0.043, 95% CI: 0.003-0.589, p = 0.018) and GTR after LMS diagnosis (HR: 0.058, 95% CI: 0.009-0.384, p = 0.003) were statistically significantly associated with longer OS, while non-adjuvant therapy after LMS diagnosis (HR: 30.58, 95% CI: 4.68-199.89, p = 0.000) was predictor of shorter OS (Table 3). The median OS in patients with pathology grade III and GTR after LMS diagnosis were longer than those with pathology grade IV, non-GTR after LMS diagnosis (31.5 vs. 15.0 months, p = 0.000; 26.0 vs. 15.0 months, p = 0.004, respectively; log-rank test; Fig. 3). The median OS in patients with non-adjuvant therapy after LMS diagnosis was shorter than patients with adjuvant therapy after LMS diagnosis (12.0 vs. 20.0 months, p = 0.001, log-rank test; Fig. 3).

**Table 3:** Overall survival by univariable and multivariable Cox analyses

Variable	N=114	Univariate			Multivariate		
		HR	95% CI	P-value	HR	95% CI	P-value
Age (<40)	60	0.702	0.447-1.032	0.072			
Gender (Male)	76	0.989	0.660-1.481	0.956			
Pathology grade III	31	0.343	0.214-0.548	0.000	0.043	0.003-0.589	0.018
KPS≥80	95	0.480	0.287-0.804	0.005			
GTR at glioma diagnosis	52	0.313	0.204-0.480	0.000			
Adjuvant therapy after glioma diagnosis							
Radiotherapy	90	0.354	0.217-0.577	0.000			
Chemotherapy	98	0.373	0.214-0.651	0.001			
Antiangiogenic therapy	24	1.011	1.625-1.634	0.966			
Intrathecal MTX	12	0.623	0.324-1.200	0.157			
Primary LMS	42	3.147	2.046-4.841	0.000			
GTR after LMS diagnosis	27	0.511	0.317-0.823	0.006	0.058	0.009-0.384	0.003
Non-adjuvant therapy after LMS diagnosis	33	2.044	1.346-3.105	0.001	30.58	4.68-199.89	0.000
IDH mutation	21	0.285	0.162-0.501	0.000			
MGMT methylation		0.220	0.062-0.788	0.020			
Yes	6						
No	20

### Prognostic factors of PLS in all patients

The median PLS was 6.0 months. Univariate analysis revealed KPS of ≥80 at LMS diagnosis (HR: 0.472, 95% CI: 0.229-0.744, p = 0.001), nodular LMS (HR: 0.468, 95% CI: 0.269-0.815, p = 0.007), MRI type Ia (HR: 0.600, 95% CI: 0.402-0.896, p = 0.012), GTR after LMS diagnosis (HR: 0.582, 95% CI: 0.365-0.927, p = 0.023), radiotherapy after LMS (HR: 0.603, 95% CI: 0.398-0.997, p = 0.048), chemotherapy after LMS (HR: 0.362, 95% CI: 0.237-0.551, p = 0.000), Intrathecal MTX (HR: 0.473, 95% CI: 0.245-0.913, p = 0.026) were associated with better PLS, while non-adjuvant therapy after LMS diagnosis (HR: 4.662, 95% CI: 2.887-7.528, p = 0.000) and MRI type II (HR: 2.217, 95% CI: 1.443-3.405, p = 0.000) were associated with shorter PLS (Table 4). Multivariable analysis revealed nodular LMS (HR: 0.530, 95% CI: 0.300-0.938, p = 0.029), GTR after LMS diagnosis (HR: 0.554, 95% CI: 0.346-0.885, p = 0.013), and non-adjuvant therapy after LMS diagnosis (HR: 4.273, 95% CI: 2.635-6.931, p = 0.000) were identified as independent prognostic factors on PLS (Table 4). The median PLS in patients with nodular LMS and GTR after LMS diagnosis were longer than those without GTR (17.0 vs. 6.0 months, p = 0.005; 9.0 vs. 6.0 months, p = 0.017, respectively; log-rank test; Fig. 3). The median PLS in patients with non-adjuvant therapy after LMS diagnosis was shorter than patients with adjuvant therapy after LMS diagnosis (3.0 vs. 8.5 months, p = 0.000, log-rank test; Fig. 3).

**Table 4:** Post-LMS survival by univariable and multivariable Cox analyses

Variable	N=114	Univariate			Multivariate		
		HR	95% CI	P-value	HR	95% CI	P-value
Age (<40) at LMS diagnosis	57	0.881	0.663-1.423	0.881			
Gender (Male)	76	1.022	0.679-1.539	0.917			
Pathology grade III	11	1.006	0.853-1.186	0.942			
KPS (≥80) at LMS diagnosis	87	0.472	0.229-0.744	0.001			
Nodular LMS	18	0.468	0.269-0.815	0.007	0.530	0.300-0.938	0.029
MRI type							
Ia	50	0.600	0.402-0.896	0.012			
Ib	27	0.948	0.759-1.185	0.693			
II	37	2.217	1.443-3.405	0.000			
Hydrocephalus	27	0.950	0.609-1.483	0.822			
GTR after LMS diagnosis	27	0.582	0.365-0.927	0.023	0.554	0.346-0.885	0.013
Adjuvant therapy after LMS diagnosis							
Radiotherapy	27	0.603	0.398-0.997	0.048			
Chemotherapy	69	0.362	0.237-0.551	0.000			
Antiangiogenic therapy	23	0.837	0.512-1.369	0.478			
Non-adjuvant therapy	33	4.662	2.887-7.528	0.000	4.273	2.635-6.931	0.000
Intrathecal MTX	12	0.473	0.245-0.913	0.026			
Primary LMS	42	0.729	0.490-1.085	0.120			
IDH mutation	21	0.689	0.406-1.170	0.168			
MGMT methylation		0.723	0.239-2.185	0.565			
Yes	6						
No	20						

### Prognostic factors of OS in GBM subgroup

**Fig. 4:**
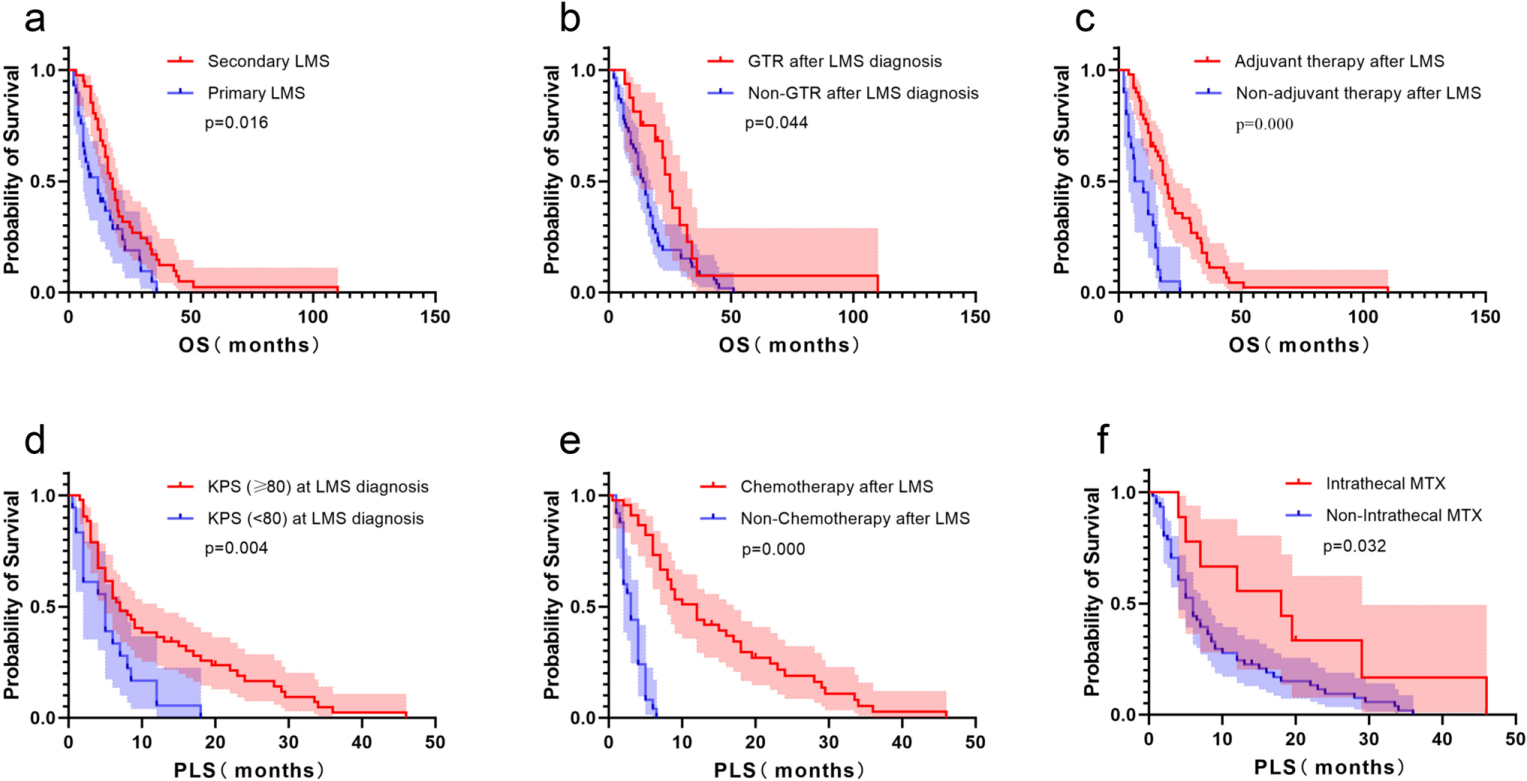
Comparison of OS and PLS by Kaplan–Meier curves in GBM subgroup. OS of (**a**) primary LMS, (**b**) GTR after LMS diagnosis and (**c**) non-adjuvant therapy after LMS diagnosis; PLS of (**d**) KPS (≥80) at LMS diagnosis, (**e**) Chemotherapy after LMS and (**f**) Intrathecal MTX

In univariable analysis, KPS of ≥80 (HR: 0.467, 95% CI: 0.230-0.946, p = 0.035), GTR at glioma diagnosis (HR: 0.449, 95% CI: 0.268-0.750, p = 0.002), radiotherapy (HR: 0.471, 95% CI: 0.258-0.859, p = 0.014), chemotherapy (HR: 0.157, 95% CI: 0.075-0.331, p = 0.000), Intrathecal MTX (HR: 0.431, 95% CI: 0.202-0.916, p = 0.029), GTR after LMS diagnosis (HR: 0.545, 95% CI: 0.295-1.004, p = 0.052), IDH mutation (HR: 0.327, 95% CI: 0.129-0.830, p = 0.019) had better survival, while primary LMS (HR: 1.837, 95% CI: 1.099-3.070, p = 0.020) and non-adjuvant therapy after LMS diagnosis (HR: 3.830, 95% CI: 2.117-6.929, p = 0.000) had shorter survival (Table 5). Multivariable analysis revealed that GTR after LMS diagnosis (HR: 0.431, 95% CI: 0.227-0.821, p = 0.010), primary LMS (HR: 4.209, 95% CI: 2.270-7.804, p = 0.000) and non-adjuvant therapy after LMS diagnosis (HR: 7.879, 95% CI: 3.821-16.245, p = 0.000) were independent prognostic factors on OS (Table 5). The median OS in patients with GTR after LMS diagnosis was longer than the patients without GTR (25.0 vs. 14.0 months, p = 0.044, log-rank test; Fig. 4). The median OS in patients with primary LMS and non-adjuvant therapy after LMS diagnosis were shorter than the others (12.0 vs. 18.0 months, p = 0.016; 6.5 vs. 19.0 months, p = 0.000, respectively, log-rank test; Fig. 4).

**Table 5:** Overall survival by univariable and multivariable Cox analyses of GBM subgroup

Variable	N=70	Univariate			Multivariate		
		HR	95% CI	P-value	HR	95% CI	P-value
Age (<40)	30	0.598	0.353-1.013	0.056			
Gender (Male)	45	0.946	0.573-1.561	0.827			
KPS≥80	60	0.467	0.230-0.946	0.035			
GTR at glioma diagnosis	27	0.449	0.268-0.750	0.002			
Adjuvant therapy after glioma diagnosis							
Radiotherapy	54	0.471	0.258-0.859	0.014			
Chemotherapy	60	0.157	0.075-0.331	0.000			
Antiangiogenic therapy	12	0.835	0.424-1.646	0.603			
Intrathecal MTX	9	0.431	0.202-0.916	0.029			
Primary LMS	29	1.837	1.099-3.070	0.020	4.209	2.270-7.804	0.000
GTR after LMS diagnosis	16	0.545	0.295-1.004	0.052	0.431	0.227-0.821	0.010
Non-adjuvant therapy after LMS diagnosis	20	3.830	2.117-6.929	0.000	7.879	3.821-16.245	0.000
IDH mutation	8/66	0.327	0.129-0.830	0.019			
MGMT methylation		0.360	0.096-1.352	0.130			
Yes	4						
No	13

### Prognostic factors of PLS in GBM subgroup

In GBM subgroup, univariate analysis demonstrated that KPS of ≥80 at LMS diagnosis (HR: 0.459, 95% CI: 0.259-0.811, p = 0.007), nodular LMS (HR: 0.481, 95% CI: 0.259-0.895, p = 0.021), MRI type II (HR: 2.061, 95% CI: 1.158-3.668, p = 0.014), GTR after LMS diagnosis (HR: 0.571, 95% CI: 0.314-1.039, p = 0.067), chemotherapy after LMS (HR: 0.106, 95% CI: 0.050-0.222, p = 0.000), Intrathecal MTX (HR: 0.441, 95% CI: 0.200-0.972, p = 0.042) and primary LMS (HR: 0.576, 95% CI: 0.350-0.948, p = 0.030) were associated with better PLS, while non-adjuvant therapy after LMS diagnosis (HR: 4.662, 95% CI: 2.887-7.528, p = 0.000) was associated with shorter PLS (Table 6). Multivariable analysis revealed that KPS of ≥80 at LMS diagnosis (HR: 0.472, 95% CI: 0.256-0.870, p = 0.016), chemotherapy after LMS (HR: 0.105, 95% CI: 0.048-0.229, p = 0.000) and Intrathecal MTX (HR: 0.382, 95% CI: 0.150-0.974, p = 0.044) were independent prognostic factors of PLS (Table 6). The median PLS in patients with KPS of ≥80 at LMS diagnosis, chemotherapy after LMS and Intrathecal MTX was longer than those opposites (7.0 vs. 5.0 months, p = 0.004; 12.0 vs. 3.0 months, p = 0.000; 18.0 vs. 6.0 months, p = 0.032, respectively, log-rank test; Fig. 4).

**Table 6:** Post-LMS survival by univariable and multivariable Cox analyses of GBM subgroup

Variable	N=70	Univariate			Multivariate		
		HR	95% CI	P-value	HR	95% CI	P-value
Age (<40) at LMS diagnosis	30	0.840	0.514-1.375	0.488			
Gender (Male)	45	1.160	0.681-1.977	0.585			
KPS (≥80) at LMS diagnosis	52	0.459	0.259-0.811	0.007	0.472	0.256-0.870	0.016
Nodular LMS	14	0.481	0.259-0.895	0.021			
MRI type							
Ia	34	0.664	0.401-1.100	0.112			
Ib	16	0.907	0.507-1.622	0.742			
II	20	2.061	1.158-3.668	0.014			
Hydrocephalus	13	0.817	0.444-1.506	0.517			
GTR after LMS diagnosis	16	0.571	0.314-1.039	0.067			
Adjuvant therapy after LMS diagnosis							
Radiotherapy	18	0.635	0.364-1.110	0.111			
Chemotherapy	45	0.106	0.050-0.222	0.000	0.105	0.048-0.229	0.000
Antiangiogenic therapy	12	0.857	0.433-1.695	0.657			
Non-adjuvant therapy	20	7.053	3.558-13.979	0.000			
Intrathecal MTX	9	0.441	0.200-0.972	0.042	0.382	0.150-0.974	0.044
Primary LMS	29	0.576	0.350-0.948	0.030			
IDH mutation	8/66	0.452	0.193-1.059	0.067			
MGMT methylation		1.489	0.456-4.866	0.510			
Yes	4						
No	13						

## Discussion

LMS is considered to entail a particularly bad prognosis and remains a late-stage manifestation [6, 13]. Previous articles have mostly focused on studying factors related to the occurrence of LMS or prognostic factors related to total OS [6, 8, 11, 13, 15–17]. For example, they demonstrated that ventricular entry or tumor contact with the subventricular zone (SVZ) might be associated with leptomeningeal dissemination [16, 17]. Park et al. reported that chemotherapy, radiotherapy combined with chemotherapy, KPS, and male patients are associated with longer OS [15]. However, the evidence of current therapeutic strategies after LMS diagnosis remains lacking, and there are no standardized treatment method or consensus after LMS diagnosis. Therefore, we bring the characteristics and treatment methods after glioma dissemination into the survival analysis and systematically investigate the prognostic factors of post-LMS survival to guide the subsequent LMS treatment. Our study revealed that the median OS of this cohort is 17.0 months, which has been corroborated in a previous study [15]. The median PLS is 6.0 months, which might be slightly longer than previous reports of 3-5 months [7, 10]. Our study also found a surprising result - GTR after LMS diagnosis and adjuvant therapy after LMS diagnosis were independent prognostic factors on OS and PLS. which might be contrary to previous findings [9, 13]. All these results suggest that surgical treatment after HGG dissemination is not as pessimistic as previously thought, and also emphasizes the importance of adjuvant treatment after dissemination. therefore, we continued further analysis.

Previous studies suggested that surgical management is not suitable, due to the multifocal character of LMS [9]. The most commonly used surgical treatment of LMS was VP shunt, because communicating hydrocephalus was correlated with the presence of LMS [18, 19]. To our knowledge, our institution reported the largest cohort of patients who underwent surgical resection after dissemination. Our findings demonstrated that GTR after LMS diagnosis was an independent prognostic factor on both OS and PLS. In this cohort, 80% patients coexist with primary or secondary tumors at the time of LMS diagnosis. So maximal safe resection of local disseminated lesions or recurrent lesions to reduce tumor volume and intracranial pressure might contribute to prolong PLS. Of course, this may also be the result of the local lesions more likely to GTR. Dardis et al. demonstrated that the time to development of LMS in patients with grade III tumors appears longer than GBM patients [8]. While there is no difference in PLS between different pathology grade in our study. This may explain the result that pathology grade III was associated with longer OS in our study, which was different from a previous study [15].

To exclude the influence of pathological grade, we conducted a GBM subgroup analysis. Similar to the results from the total cohort, GTR after LMS diagnosis and non-adjuvant therapy after LMS diagnosis are independent factors of OS. Multivariable analysis also revealed that the median OS of patients with primary LMS were shorter than secondary LMS, which is corroborated in previous studies [7, 15]. Among the GBM subgroup, Multivariable analysis also revealed chemotherapy after LMS was an independent prognostic factor of PLS. The effect of chemotherapy is obvious and has been confirmed in several previous studies [8, 10, 15]. In addition to chemotherapy, intrathecal MTX is also one of the important treatments [12]. Noh et al. revealed the median survival after LMS diagnosis in the Intrathecal MTX treatment group was longer than that in the conservative management group, but not longer than that in other treatment group [12]. However, Intrathecal MTX treatment has been confirmed as an independent prognostic factor of PLS in our study. Several studies demonstrated that intrathecal MTX in combination with systemic chemotherapy is a potentially effective therapy for patients with LMS [20–22]. It suggested that chemotherapy and Intrathecal MTX treatment are feasible treatments after dissemination, but there is need to validate this by prospective research.

Patients with higher KPS had a longer OS [6], which has been corroborated in our results in both total cohort and subgroup analysis. But it was not an independent prognostic factor of OS. Dardis et al. reported that higher KPS at LMS diagnosis was associated with longer OS [8]. Our study has reached another interesting result that KPS at LMS diagnosis was an independent prognostic factor of PLS. Patients with higher KPS at LMS diagnosis may receive more treatments, which might contribute to a longer OS and PLS.

Previous studies have defined LMS as two types: disseminated LMS and subependymal LMS [7, 15]. However, OS and PLS revealed no significant difference between the two types [7, 11]. A mixed pattern has also been observed. Therefore, we introduced a new classification pattern. Univariate results revealed that MRI type II is an unfavorable independent prognostic factor with PLS in both total cohort and subgroup. It indicated that the prognosis was worse when both subventricular and subarachnoid spaces are disseminated simultaneously. Our data also imply that patients with nodular LMS have a longer PLS, suggesting the extent of tumor dissemination affecting the survival after LMS. Our study revealed no difference between local recurrence +LMS and simple LMS, which was contrary to our initial expectation. In this study, fifty-five (76.4%) cases in the secondary LMS group had ventricular entry during resection. However, the association between ventricular entry during the initial surgery and LMS is controversial. Akshitkumar et al. demonstrated that SVZ-but not ventricular entry-associated with LMS and hydrocephalus [16]. However, a recent study found that ventricular entry is associated with LMS in GBM patients [17].

This study diagnosed 27 cases with hydrocephalus, and all patients underwent V-P shunt. Kim et al. reported that the hydrocephalus treatment with a V-P shunt in patients with LMS could improve symptoms and prolong OS [23]. However, our study found no significant difference in PLS between patients with or without hydrocephalus. It indicates that hydrocephalus does not affect patient survival, as most hydrocephalus can usually be resolved by V-P shunt. This single-center retrospective study has inevitable limitations. First, this is not a randomized controlled trial. So, these results look promising but should be interpreted with caution. Second, this study is unable to obtain more molecular indicators. Third, the understanding of LMS is gradually deepening because of the longtime span, thus the incidence of LMS in the study may be lower than the actual incidence rate.

## Conclusion

Safe gross total resection of tumor and subsequent adjuvant therapy after leptomeningeal spread remains a powerful weapon to improve survival for HGG patients with LMS. Chemotherapy and Intrathecal MTX treatment are feasible treatment options after LMS and might improve OS. The extent of tumor dissemination may affect the survival after LMS. The treatment of patients with glioma after LMS diagnosis is very challenging and limited. Therefore, prospective studies and clinical trials are greatly needed to find an effective, systematic treatment approach.

## Data Availability

The datasets generated during the current study are available from the corresponding author on reasonable request.
